# Impact of Leaf Removal on Phenolics and Antioxidant Activity of Trebbiano Berries (*Vitis vinifera* L.)

**DOI:** 10.3390/plants11101303

**Published:** 2022-05-13

**Authors:** Mike Frank Quartacci, Cristina Sgherri, Calogero Pinzino

**Affiliations:** 1 Department of Agriculture, Food and Environment, University of Pisa, 56124 Pisa, Italy; cristina.sgherri@unipi.it; 2 Institute of Chemistry of Organometallic Compounds (ICCOM-CNR), Italian National Research Council, 56124 Pisa, Italy; rino@pi.iccom.cnr.it

**Keywords:** antioxidant activity, ascorbate, defoliation, electron paramagnetic resonance, flavonols, grape, phenolic acids, phenols, Trebbiano

## Abstract

Leaf removal is a canopy management practice widely applied in viticulture to enhance the phenol composition and concentration of grapes, which then results in improved wine quality. Many studies were carried out on red berried varieties, but information on white ones is scanty. The aim of the study was to assess the effect of basal leaf defoliation in post fruit set on the phenol composition, ascorbate level and antioxidant activity of Trebbiano grapes. Electron paramagnetic resonance was also employed to monitor the decay kinetics of 1,1-diphenyl-2-picrylhydrazyl which allowed the identification of antioxidants with different action rates. The results show that defoliation caused an increase in the phenolic acid (hydroxycinnamic and hydroxybenzoic acids) and flavonol concentrations of berries without changes in the composition. Both ascorbate and antioxidant activity were also enhanced in the berries from defoliated vines. Besides increasing the number of fast-rate antioxidants, leaf removal resulted in the formation of intermediate-rate ones. In the Trebbiano variety, leaf removal in the post fruit set may represent an effective strategy to enhance the phenolic composition and the antioxidant defense system of berries.

## 1. Introduction

Among the biologically active metabolites that contribute to the defense against environmental adversities, phenols play a particularly important role. In fact, phenolic compounds represent one of the main constituents of the antioxidative defenses of cells. The antioxidative action of this group of compounds is linked to their ability to avoid the beginning or to slow down the propagation of cell oxidation which causes cell damage due to lipid peroxidation and enzyme inactivation [1]. The phenolic classes detected in grapes are represented by flavonoids (flavonols, anthocyanins, flavan-3-ols and their polymeric form proanthocyanidins) and non-flavonoids (benzoic and hydroxycynnamic acid derivatives). The relative amount and distribution of these compounds depend on a variety of factors such as grape variety, vineyard location, climate, soil type, cultivation practices (among which canopy management and irrigation) and harvesting time [2]. Phenolic hydroxycinnamates are commonly accumulated in berry skin and especially in the flesh and are usually the most abundant class of phenolics in white berries, *p*-coumaric, caffeic and ferulic acids being the main constituents [3]. The hydroxybenzoic acid concentration is lower than that of hydroxycinnamic acids and gallic acid is the most abundant component. Flavonol synthesis occurs primarily during the early stages of fruit development in the outer epidermis of the skin, being sunscreen protectors, and ends at around *veraison* [4]. The main representatives of this class are kaempferol, quercetin, and myricetin (and its methylated form isorhamnetin). In cells, phenolic compounds may be present in both free and conjugated forms, and their chemical structure has a significant impact on their bioavailability and protective action. It has been found that the degree of hydroxylation of phenolics and the relative position of the hydroxyl groups remarkably affect the antioxidant capacity of the individual compounds [5].

Epidemiological and clinical studies have evidenced that the intake of grape and its derivatives is closely related to the prevention of many human diseases associated with oxidative stress due to the presence of a variety of antioxidants among which phenols. [6,7]. Together with phenols, ascorbate also plays a fundamental role in countering the formation of reactive oxygen species (ROS), either directly or through the glutathione-ascorbate cycle. Ascorbate occurs in a reduced form (AsA) and two oxidized forms (mono- and dehydroascorbate). The ratio between reduced and oxidized ascorbate is essential for the ability of the plant to fight oxidative stress. It is well-known that red grape varieties contain more phenolic compounds than white ones. However, epidemiological as well as in vitro studies suggest that white grapes and wines can have the same health benefits as the red varieties [8]. Studies carried out on grape berries and wines [9,10] correlated their overall antioxidant power to the total amount of polyphenols via electron paramagnetic resonance (EPR) determination. However, there is little information about the interactions within the single classes of compounds. Using the EPR technique, it is possible to distinguish among phenolics slow-, intermediate- and fast-rate antioxidants. Pérez-Lόpez and co-workers observed that flavonoids (quercetin) and anthocyanins accounted for the majority of the last two types of antioxidants, respectively [1].

Leaf removal (defoliation) is a grape canopy management practice widely used—from flowering (early defoliation) to fruit set until *veraison* (traditional defoliation)—to enhance canopy microclimate due to improved air circulation and light penetration [8,11,12]. As a result, grapes well-exposed to sunlight have higher sugar, anthocyanin, and phenolic concentrations than shaded grapes. The photo-regulation of the invertase and phenylalanine ammonia lyase enzymes are thought to be primarily involved in these responses to leaf removal [13]. It has been observed that regardless of the period and method of defoliation (manual or mechanical), leaf removal led to the accumulation of flavonols and anthocyanins in Tempranillo grapes due to an increase in total leaf area per yield [14]. Early defoliation also decreased cluster compactness and yield, but increased total phenolics, anthocyanin and tannin concentrations in both berries [15,16,17] and wines [18,19]. Non-flavonoid and flavonoid biosynthetic pathways are subjected to the action of regulatory enzymes that are light- and temperature-sensitive [4]. Thus, any change in the canopy microclimate, such as those caused by leaf removal, may have a remarkable effect on the synthesis and accumulation of these compounds in berries and on wine quality.

Studies concerning the effect of defoliation on the phenolic composition and the antioxidant power of white grapes are very few compared to red berried grapes due to their lower phenolic content. Among white grapes, Trebbiano Toscano is certainly one of the most important varieties of the great Trebbiano family. It is grown mainly in Tuscany and Umbria to produce wines with a savory and fresh taste which are dry and rather acidic. As defoliation is an effective management practice for improving the synthesis of secondary compounds, this study aimed to evaluate how leaf removal, carried out in post-fruit setting, can influence Trebbiano grape characteristics evaluating the phenolic composition, the antioxidant capacity and the rate at which antioxidants exert their action in berries.

## 2. Results

### 2.1. Total Phenols, Antioxidant Activity and Ascorbate

**Figure 1:**
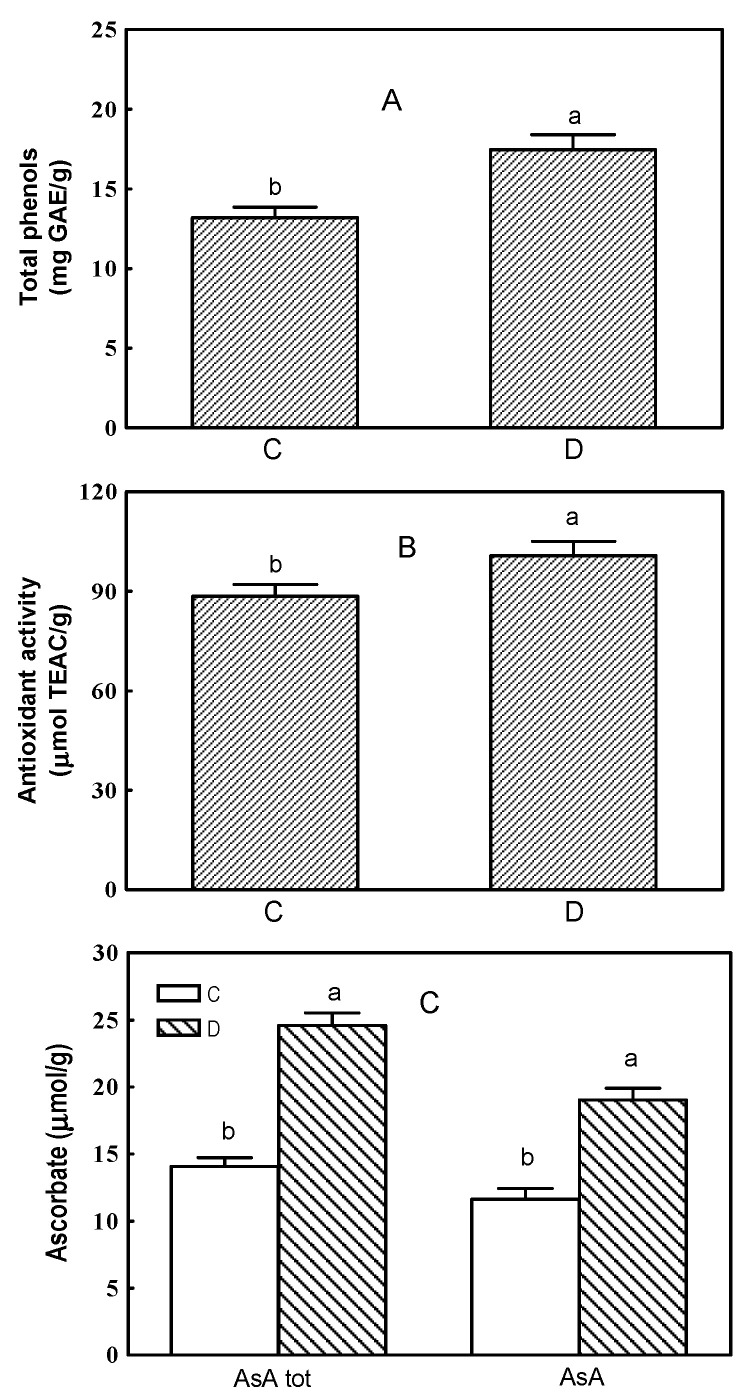
Total phenols (**A**), antioxidant activity (**B**) and ascorbate (**C**) in grape berries from control and defoliated vines of *Vitis vinifera* (Trebbiano variety). C, control; D, defoliated; AsA, reduced ascorbate. Data are reported as mean values ± standard error. For each compound means accompanied by different letters are significantly different at *p* ≤ 0.05.

Leaf removal in post fruit set enhanced the total phenol concentration of grape berries, which increased from 13.2 mg in the control to 17.5 mg GAE (gallic acid equivalents) g^−1^ fresh weight in the defoliated vines (Figure 1A). Also, the total antioxidant activity increased due to defoliation, even though to a lesser extent (Figure 1B). Total ascorbate and AsA concentrations were positively affected by the treatment resulting in a 63 and 75% increase, respectively, compared to the controls (Figure 1C). However, following the removal of leaves the percentage of AsA out of the total remained almost constant (83 and 78% of total ascorbate in the control and in the berries from defoliated vines, respectively).

### 2.2. Phenolic Acids and Flavonols

As reported in Table 1, the total free phenolic acid concentration of grape berries was more than doubled by defoliation (2.4-fold enhancement) showing values ranging from 330 to 794 μg g^−1^ fresh weight. Chlorogenic acid was by far the most abundant free phenolic acid in berries (about 97% of total), and its concentration increased from 320 to 771 μg g^−1^ fresh weight after leaf removal (Table 1). Protocatechuic, vanillic, syringic, ferulic, and *p*-coumaric acids represented the other detected free phenolic acids, *p*-coumaric acid being the only one that suffered a reduction following defoliation.

The concentration of total conjugated phenolic acids in grape berries showed the same trend of free phenolic acids (Table 2). Indeed, also in this case the treatment resulted in an enhancement of total phenolic acids, although of reduced entity (1.3-fold). Caffeic acid, which is the product of chlorogenic acid hydrolysis, represented the main phenolic acid (64% on average), followed by *p*-coumaric (29%) and gallic acids (6%). Even if the concentrations of the main conjugated phenolic compounds increased after defoliation, their percentages did not change significantly.

Leaf removal increased the concentration of flavonols by 1.8-fold compared to the control (Table 3) as well as those of the main components, namely quercetin glucuronide (2.0-fold), quercetin glucoside (+52%) and kaempferol glucoside (+85%). The different forms of isorhamnetin (phenols with the lowest concentrations) did not show any change following defoliation.

### 2.3. Electron Paramagnetic Resonance

**Figure 2:**
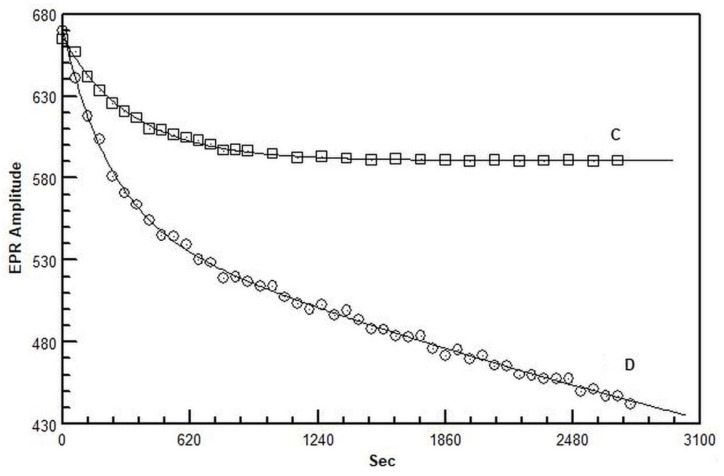
EPR decay kinetics of 1,1-diphenyl-2-picrylhydrazyl (DPPH•) obtained from berry extracts from control and defoliated vines of *Vitis vinifera* (Trebbiano variety). C, control; D, defoliated.

The EPR spectrum of the 1,1-diphenyl-2-picrylhydrazyl (DPPH•) radical looks like a narrow five-line shape which allows the recording of decay kinetics with very close points. DPPH• shows a well-resolved quintet EPR spectrum having at *g* = 2.0036 *a*_N1_ and *a*_N2_ values of 0.927 and 0.846 mT, respectively, and a unimolecular decay constant of 2.06 × 10^−6^ s^−1^ [20,21]. The DPPH• antioxidant assay is commonly used to quantify the antioxidant capacity of a compound, the radical reactions involved during the scavenging being well described [22]. The DPPH• decay kinetics following the addition of grape berry methanolic extracts of both control and defoliated vines are reported in Figure 2.

The decay kinetics show the contribution of one or two pseudo-first-order kinetics, depending on the treatment: one for the control and two for the defoliated plants. The pseudo-first-order kinetics are associated with antioxidants or groups of antioxidants of different nature, each having a different scavenging rate as evidenced by the rate constants (*k*) which indicate the speed of DPPH• disappearance [22]. The control berries were characterized by a fast rate constant, whereas those collected from defoliated vines showed both fast and intermediate rate constants (Table 4).

The decay rate constants *k*, which characterize the decay kinetics of DPPH•, are indicative of the presence in the extracts of antioxidants having a fast and intermediate antioxidant action. The antioxidant power evaluated by EPR indicates the ability of 1 g of grape berry to reduce DPPH• molecules in the assay. A significant increase in the fast antioxidant constant rate and number was observed in the extracts of defoliated berries (Table 4). Moreover, the defoliation treatment resulted in the appearance of intermediate rate antioxidants that were not detectable in the control one. Consequently, the number of the fast and intermediate antioxidants calculated by the reduced DPPH• molecules also followed the same trend.

## 3. Discussion

In our study, the removal of leaves in the post fruit set stage caused an increase in the concentration of total phenols, phenolic acids and flavonols. A similar increase has previously been observed by other authors in white and red grape varieties [12,17,18,23]. This increase is due to higher temperatures from increased sunlight after defoliation. There is a general agreement on the positive effect of sunlight on phenol accumulation in berries [14]. Indeed, leaf removal in the cluster zone may affect the synthesis of phenolic compounds in grapes due to an increased exposure to UV radiation [24]. The biosynthetic pathway of phenolic substances is regulated by enzymes that are light and temperature dependent. Hence, the changes in microclimatic conditions such as those caused by defoliation may have a remarkable effect on the synthesis and accumulation of these substances [14]. Furthermore, it should not be forgotten that changes in the seasonal climatic conditions during grape ripening among different years may have a significant influence on leaf removal efficiency and phenol accumulation.

The increase in UV-absorbing compounds such as hydroxycinnamic acids and flavonoids is a response aimed at protecting cell membranes as an increased level of these phenolics is correlated with a more efficient absorption of the harmful UV radiation [25]. Phenolics also enhance protection against photooxidative stress as they have chemical structures capable of scavenging free radicals due to their hydroxylic groups, the number and position of which determine their antioxidant capacity [26]. As the synthesis of hydroxycinnamates occurs mainly before *veraison* [3] and is light-dependent, the defoliation treatment itself and the consequent higher foliage lighting could also have caused an increase in their concentration.

The defoliation treatment increased the concentration of both free and conjugated hydroxycinnamic and hydroxybenzoic acids but did not alter the percentage of the individual compounds within each class. This is probably due to an increase in the rate of synthesis of these compounds induced by the enhanced UV radiation in the canopy zone of the defoliated vines and/or to a greater size and weight of the berries (data not shown). Bubola et al. [18] suggested that the effect of light dominates over the effect of temperature in the enhancement of hydroxycinnamates based on the striking differences in PAR values between control and defoliated vines. Leaf removal did not alter the qualitative composition of phenolic acids suggesting that enzymes involved in the biosynthetic pathway were affected only in their activity and not in their expression.

A higher synthesis of flavonols in grapes due to increased sun exposure caused by defoliation has widely been reported by several authors [12,23,27]. Indeed, the concentration of flavonols can be considered as an index to assess canopy architecture and the exposure of grapes to solar radiation within the canopies following microclimate management induced by defoliation [28].

The increase in the concentration of phenols following defoliation resulted in an enhanced antioxidant activity of the extracts. Indeed, it is known that phenols are one of the main components of the cellular antioxidant defense mechanism. Our results agree with the study by Radovanovic et al. [29] in which early defoliation of vines resulted in a significant increase in total phenol concentration as well as in antioxidant activity compared to the control. Pavic et al. [30] observed a similar trend in the grape skins of the Merlot variety following leaf removal just after blooming. It has been observed that the antioxidant capacity differs among phenols being expressed at different levels, making it difficult to differentiate the relative contribution of the various phenolics to the total antioxidant capacity of berries. The antioxidant power of phenolic acids and flavonoids depends on the number of hydroxyl and methoxyl groups present on the phenyl rings [26]. The about 2-fold increase in the levels of quercetin derivatives and chlorogenic acid in berries from defoliated vines could explain the higher antioxidant capacity as suggested by Perez-Lopez et al. [1] who observed that the DPPH• scavenging ability was strictly related with the presence of both compounds in lettuce extracts.

Due to their potential toxicity to cell structures [31], about 80% (control) and 70% (defoliated) of the grape phenolic acids were in the conjugated form (Table 2). Following the observation that the antioxidant capacity of free phenolic acids was greater than that of bound phenolics [32], it may be suggested that the enhanced antioxidant capacity of berries from defoliated vines could be also linked to a reduced presence of the conjugated forms.

Among antioxidants, ascorbate plays a key role being involved in ROS detoxification due to its strong reducing properties that allows the neutralization of ROS and the reduction of molecules oxidized by ROS in cooperation with glutathione in the Foyer-Halliwell-Asada cycle [33]. The increased total ascorbate concentration in the berries following defoliation suggests a de novo synthesis to counteract the possible damaging effects of the more intense irradiation reaching the bunches. It should be noted that the percentage of AsA did not change in the two treatments compared to the total ascorbate concentration, demonstrating that the cellular oxidative status was maintained or that no serious stressful events occurred and AsA was not consumed. The higher level of phenols and antioxidant activity of berries from defoliated vines is particularly important for what concerns the health and nutraceutical properties of the berry themselves and their by-products, primarily wine [34]. Indeed, epidemiological studies suggested that a high and continuous consumption of foods rich in plant polyphenols provides some protection against the occurrence of cancers, cardiovascular diseases, diabetes, insulin resistance, obesity, neurodegenerative diseases, and osteoporosis [35]. Thus, defoliation may represent a powerful and simple agronomic technique to upregulate flavonoid biosynthesis in grape berries and consequently increase the antioxidant protection against toxic reactive species.

The addition of the DPPH• radical to the extracts and its low decay constant allowed the determination of the antioxidant power and the kinetic behavior of the antioxidants present in berries. The decay rate constants are indicative of the presence of two groups of protective compounds: fast- and intermediate-rate antioxidants. The fast-rate ones were identified in both control and defoliated treatments, whereas the intermediate-rate antioxidants were detected only in berries collected after leaf removal. The highest *k*_F_ value and the number of antioxidants of the defoliation treatment compared to the control (+44 and 47%, respectively) may be mainly related to the enhanced phenol concentration (+32%) and to a lesser extent to ascorbate. The absence of significant changes in the phenolic percentage composition of the various classes suggests that defoliation might have caused the increase of specific scavenging molecules (phenolic and non-phenolic) not identified in this study and belonging to the intermediate-rate antioxidants.

## 4. Materials and Methods

### 4.1. Chemicals

All chemicals, reagents, and phenolic acid standards used in this study were analytical or HPLC grade from Sigma-Aldrich (Milan, Italy). Water was of Milli Q grade. Flavonol standards were purchased from Extrasynthèse (Genay, France).

### 4.2. Experimental Setup

The vineyard was in the province of Pisa (43°4′41″52 N; 10°40′44′76 E). The vines (*Vitis vinifera* L., Trebbiano variety), grafted onto a 1103P rootstock and planted in north-south oriented rows spaced 2.5 × 1 m, were grown under field conditions, and were trained to a single curtain cordon. The experimental area was about 1 m above mean sea level on flat land and was characterized by a Typic Xeropsamment loamy soil (44% sand, 34% silt and 22% clay) with a low level of soil organic matter (1.61%) and pH 8.4. The climatic conditions were typical of the Mediterranean, with a mean yearly air temperature of 15 °C.

The trial was set on a randomized block design with three blocks per treatment (with each row as a block), with each block containing 10 vines. Defoliation was carried out manually in post-fruit setting on 2 July 2020 by removing the six basal leaves (100% basal leaf removal) of all of the primary vine shoots. Healthy grape berries from both groups were sampled on 5 October 2020 at the technological maturity stage. Sampling was carried out by random berry picking from selected and healthy bunches. Every sample was taken in three repetitions (from three blocks) each comprising 20 berries from various parts of bunches. Grape berries were first weighted and immediately after fixed in liquid nitrogen, transferred to the Agricultural Chemistry section of the Department of Agriculture, Food and Environment of the University of Pisa, and stored at −80 °C. Frozen samples were then freeze-dried and reduced to a fine powder.

### 4.3. Ascorbate

After freeze-drying grape berry powders were homogenized in ice-cold 5% (*w*/*v*) trichloroacetic acid containing 4% (*w*/*v*) polyvinylpolypyrrolidone. Total ascorbate and AsA were detected in the supernatant according to Wang et al. [36]. Total ascorbate (AsA + dehydroascorbate) was determined by reducing dehydroascorbate to AsA with 0.97 mM dithiothreitol, whereas dehydroascorbate was estimated as the difference between total ascorbate and AsA concentrations. For quantification, two distinct calibration curves for AsA and total ascorbate (5–50 nmol range) were used.

### 4.4. Sample Extracts

Samples were extracted with a cold mortar and pestle under dark conditions with 70% methanol containing 1% HCl, and then sonicated for 30 min. After centrifugation at 12,100× *g* for 20 min at 4 °C, the pellets were sonicated again. Supernatants were pooled together and filtered using 0.45 μm Minisart filters (Sartorius, Goettingen, Germany).

### 4.5. Total Phenols

Total phenol concentrations were determined on methanolic extracts as described by Nguyen and Niemeyer [37] exploiting the reaction between phenols and the Folin-Ciocalteu reagent. For phenol quantification a calibration curve prepared with gallic acid was used. Total phenols were expressed as GAE g^−1^ fresh weight.

### 4.6. Antioxidant Activity

The antioxidant activity assay of the extracts was carried out following the generation of the stable radical cation ABTS•^+^ (2,2′-azino-di-[3-ethylbenzthiazoline sulphonate]) following the procedure reported by Pellegrini et al. [38]. The radical solution was diluted in ethanol to obtain an absorbance of 0.70 ± 0.05 at 734 nm. The extracts were added to the radical solution and after 10 min the reduction of absorbance was recorded and compared to that obtained using a Trolox standard. During this reaction, the blue ABTS radical cation is converted back to its colorless neutral form. Quantification of antioxidant activities was carried out using a Trolox dose-response curve in the 0.2–1.5 mM range. The antioxidant activity of grape berries was expressed as Trolox equivalent antioxidant capacity (TEAC) g^−1^ fresh weight.

### 4.7. Phenolic Acids

Phenolic acid composition was determined by RP-HPLC as described by Quartacci et al. [39]. Briefly, aliquots of methanolic extracts were injected into a Waters model 515 HPLC system (Waters, Milan, Italy) equipped with a 4.6 mm × 250 mm Prodigy ODS column (Phenomenex, Bologna, Italy) and a Waters 2487 dual λ UV-VIS detector (detection at 280 nm). Mobile phase A consisted of water and acetic acid (98 and 2%, respectively), whereas mobile phase B by water, acetonitrile, and acetic acid (68, 30 and 2%, respectively). A linear gradient of 10–90% mobile phase B was run for 60 min at 1 mL min^−1^. Identification and quantification of phenols was carried out using standards, alone or mixed, in the 0.1–0.5 µg range. Chromatogram analysis was carried out using the Millennium 32 software (Waters).

The composition of the esterified phenolic acids was determined following alkaline hydrolysis of the extracts and subsequent RP-HPLC analysis. Alkaline hydrolysis was carried out in the dark for 1 h under nitrogen after 4 N NaOH containing 1% AsA and 10 mM ethylenediaminetetraacetic acid (to prevent phenolic acid degradation) has been added to the extracts. After acidification with 12 N HCl to reach a pH value of about 2 samples were extracted three times with 1 mL ethyl acetate. The organic phases were collected following centrifugation at 3000× *g* for 5 min and taken to dryness. Immediately before analyses, the dry material was re-dissolved in 50% (*v*/*v*) acetonitrile and filtered by a Sartorius Minisart 0.45 µm filter. Hydrolyzed phenolic acid composition was determined by the RP-HPLC method described above.

### 4.8. Flavonoids

The chromatographic analysis of flavonoids was similar to the one described for phenolic acids with detection at 360 nm. Mobile phase A was constituted by water acidified with formic acid (pH 2.7), and mobile phase B by methanol. A linear gradient of 10–90% mobile phase B was run for 35 min at 1 mL min^−1^. For flavonoid identification and quantification co-chromatography of standard flavonoid mixtures in the range of 10–500 ng was used.

### 4.9. EPR Decay Kinetics

The antioxidant capacity of berry extracts was determined by recording the decay kinetics of the stable radical 1,1-diphenyl-2-picrylhydrazyl (DPPH•). For the recording of spectra, a Varian E112 (X-band) EPR spectrometer was used [20]. Spectra acquisitions were carried out in the dark to ensure that there were no photochemical effects on both DPPH• and berry extracts. Simulations of the acquired EPR spectra were carried out using the Winsim program [40], and data analyses were performed using the CurveExpert software (version 1.34; Hyams Development, Madison, AL, USA). The final concentration of DPPH• in ethanol was 400 μM. Based on preliminary experiments, this concentration was chosen being much higher than the initial concentration of antioxidants in the sample, thus allowing all of the antioxidants to react with the radical [22]. To evaluate the actual antioxidant capacity of berry extracts, decay kinetics of DPPH• without the extract were also recorded. The amplitude of the central line of the EPR spectrum was taken as a reference for recording the decay kinetics. The spectra acquisition started immediately after the addition of the extract to the DPPH• solution, and the scan time was set to 1 min. Both *k* value and number of reduced DPPH• were calculated by fitting of the kinetic curves. Antioxidant capacity was a function of the number of DPPH• molecules reduced by 1 g of berry powder and the EPR decay rate constant (M^−1^ s^−1^).

The experimental kinetic data fitted with the following equation:

A = A_I_ exp(−k_I_′ t) + A_F_ exp(−k_F_′ t) + A_R_ (1)

where A is DPPH• molar concentration at time t, A_I_, and A_F_ are the initial DPPH• molar concentrations that can be reduced by intermediate and fast antioxidant fractions, respectively, and A_R_ is the remaining DPPH• concentration in the medium as a result of antioxidant depletion. k_I_, and k_F_ represent the rate constants of the intermediate, and fast fractions, respectively, and were calculated using the following equations:

k_I_ = k_I_′/[DPPH]_t0_ (2)

k_F_ = k_F_′/[DPPH]_t0_ (3)

Equation (1) was the result of more than one differential equation of second order assuming that the initial molar concentration of DPPH• ([DPPH]_t0_) was far in excess than the molar concentration of antioxidants in the reactions [22].

### 4.10. Statistical Analysis

Data were analyzed using the Costat 6.4 program (CoHort software, Birmingham, UK). The effects of leaf removal were evaluated by one-way ANOVA and significant differences between means were assessed by the least significant difference test at 95% confidence level (*p* ≤ 0.05). Bars shown in Figures represent the standard error of the means of three independent experiments (*n* = 3) each analyzed twice.

## Figures and Tables

**Table 1:** Free phenolic acids (µg g^−1^ fresh weight) in grape berries from control and defoliated vines of *Vitis vinifera* (Trebbiano variety). Data are reported as mean values ± standard error. For each compound means accompanied by different letters are significantly different at *p* ≤ 0.05.

Phenolic Acid	Control	Defoliated
Protocatechuic acid	0.5 ± 0.09 b	2.2 ± 0.5 a
Chlorogenic acid	320.0 ± 8.8 b	771.1 ± 11.0 a
Vanillic acid	1.6 ± 0.4 b	11.6 ± 0.9 a
Syringic acid	4.8 ± 0.9 b	7.5 ± 0.2 a
*p*-Coumaric acid	2.4 ± 0.5 a	0.3 ± 0.03 b
Ferulic acid	0.6 ± 0.1 b	1.3 ± 0.3 a
Total	329.9 ± 12.0 b	794.0 ± 12.7 a

**Table 2:** Conjugated phenolic acids (µg g^−1^ fresh weight) in grape berries from control and defoliated vines of *Vitis vinifera* (Trebbiano varieties). Data are reported as mean values ± standard error. For each compound means accompanied by different letters are significantly different at *p* ≤ 0.05.

Phenolic Acid	Control	Defoliated
Gallic acid	85.1 ± 3.1 b	130.0 ± 4.3 a
Protocatechuic acid	1.2 ± 0.1 a	0.9 ± 0.1 a
*p*-Hydroxybenzoic acid	5.4 ± 0.5 a	5.2 ± 0.1 a
Caffeic acid	952.1 ± 12.6 b	1211.0 ± 15.9 a
Syringic acid	16.2 ± 1.9 a	15.5 ± 1.0 a
*p*-Coumaric acid	392.0 ± 9.1 b	596.1 ± 10.3 a
Ferulic acid	0.7 ± 0.1 a	0.2 ± 0.1 b
Total	1452.7 ± 15.86 a	1958.9 ± 20.58 b

**Table 3:** Favonols (µg/g^−1^ fresh weight) in grape berries from control and defoliated vines of *Vitis vinifera* (Trebbiano variety). Data are reported as mean values ± standard error. For each compound means accompanied by different letters are significantly different at *p* ≤ 0.05.

Flavonol	Control	Defoliated
Quercetin glucuronide	134.77 ± 14.17 b	275.16 ± 1.20 a
Quercetin glucoside	130.06 ± 19.64 b	198.46 ± 3.76 a
Kaempferol galactoside	9.96 ± 2.73 b	19.04 ± 0.54 a
Kaempferol glucuronide	3.71 ± 0.51 b	8.64 ± 0.56 a
Kaempferol glucoside	36.06 ± 7.33 b	66.76 ± 1.61 a
Isorhamnetin galactoside	2.00 ± 0.60 a	2.41 ± 0.08 a
Isorhamnetin glucoside	10.32 ± 3.06 a	12.29 ± 0.50 a
Isorhamnetin glucuronide	0.21 ± 0.11 b	0.72 ± 0.07 a
Total	327.09 ± 18.76 b	583.48 ± 4.88 a

**Table 4:** Decay rate constants (M^−1^ s^−1^) and number of antioxidants (no. reduced DPPH• molecules × 10^19^ g^−1^) obtained from EPR decay kinetics of 3.3 mM 1,1-diphenyl-2-picrylhydrazyl (DPPH) after the addition of methanolic extracts of grape berries from control and defoliated *Vitis vinifera* L. (Trebbiano variety). Data are reported as mean values ± standard error. Means within a column accompanied by different letters are significantly different at *p* ≤ 0.05.

	Decay Rate Constant		No. of Antioxidants	
	*k*_F_	*k*_I_	FRA	IRA
Control	7.47 ± 0.09 b	nd	14.00 ± 0.71 b	nd
Defoliated	10.82 ± 0.11 a	0.32 ± 0.04	20.67 ± 0.92 a	65.54 ± 1.25

*k*_F_, fast rate constant; *k*_I_, intermediate rate constant; FRA, fast rate antioxidants; IRA, intermediate rate antioxidants; nd, not detectable.

## Data Availability

The data presented in this study are available upon request from the corresponding author.
